# Distinct Roles of CK2- and AKT-Mediated NF-κB Phosphorylations in Clasmatodendrosis (Autophagic Astroglial Death) within the Hippocampus of Chronic Epilepsy Rats

**DOI:** 10.3390/antiox12051020

**Published:** 2023-04-28

**Authors:** Ji-Eun Kim, Duk-Shin Lee, Tae-Hyun Kim, Hana Park, Tae-Cheon Kang

**Affiliations:** Department of Anatomy and Neurobiology and Institute of Epilepsy Research, College of Medicine, Hallym University, Chuncheon 24252, Republic of Koreahyun1028@hallym.ac.kr (T.-H.K.);

**Keywords:** 3CAI, astrocyte, autophagy, GPx1, NAC, oxidative stress, seizure, TMCB

## Abstract

The downregulation of glutathione peroxidase-1 (GPx1) plays a role in clasmatodendrosis (an autophagic astroglial death) in the hippocampus of chronic epilepsy rats. Furthermore, N-acetylcysteine (NAC, a GSH precursor) restores GPx1 expression in clasmatodendritic astrocytes and alleviates this autophagic astroglial death, independent of nuclear factor erythroid-2-related factor 2 (Nrf2) activity. However, the regulatory signal pathways of these phenomena have not been fully explored. In the present study, NAC attenuated clasmatodendrosis by alleviating GPx1 downregulation, casein kinase 2 (CK2)-mediated nuclear factor-κB (NF-κB) serine (S) 529 and AKT-mediated NF-κB S536 phosphorylations. 2-[4,5,6,7-Tetrabromo-2-(dimethylamino)-1H-benzo[d]imidazole-1-yl]acetic acid (TMCB; a selective CK2 inhibitor) relieved clasmatodendritic degeneration and GPx1 downregulation concomitant with the decreased NF-κB S529 and AKT S473 phosphorylations. In contrast, AKT inhibition by 3-chloroacetyl-indole (3CAI) ameliorated clasmatodendrosis and NF-κB S536 phosphorylation, while it did not affect GPx1 downregulation and CK2 tyrosine (Y) 255 and NF-κB S529 phosphorylations. Therefore, these findings suggest that seizure-induced oxidative stress may diminish GPx1 expression by increasing CK2-mediated NF-κB S529 phosphorylation, which would subsequently enhance AKT-mediated NF-κB S536 phosphorylation leading to autophagic astroglial degeneration.

## 1. Introduction

Clasmatodendrosis is an autophagic and non-apoptotic type II programmed death in astrocytes. Clasmatodendritic degeneration is characterized by lysosome-derived vacuolization in hypertrophic cell body and fragmentation/vanishing of processes [1,2,3]. Clasmatodendrosis was first reported by Alzheimer and termed by Cajal [4,5]. Clasmatodendrosis is detected in aging and various pathophysiological conditions in ischemia [6], acidosis [7], dementia [6,8,9], head trauma [10], infection [11] and demyelination disease [12]. In epilepsy rats, clasmatodendritic degeneration is restrictedly detected in astrocytes within the stratum radiatum of the CA1 region (CA1 astrocytes) [13,14,15]. Although the roles of these astroglial degenerations in pathogenesis of various neurological diseases remain an open issue, clasmatodendrosis influences the duration of spontaneous seizures in chronic epilepsy rats [14,16].

The underlying mechanisms of clasmatodendrosis are relevant to oxidative stress, the impaired ATP production induced by acidosis and/or energy-consuming events, aberrant chaperone accumulation and neuroinflammation [7,13,17], although the regulatory signal pathways are largely unknown. Interestingly, nuclear factor-κB (NF-κB) is one of the upstream molecules evoking autophagic cell death of astrocytes [18,19]. Indeed, tumor necrosis factor-α (TNF-α) neutralization alleviates clasmatodendrosis by inhibiting NF-κB serine (S) 529 phosphorylation [2]. Furthermore, AKT and its downstream effector glycogen synthase kinase-3β (GSK-3β) also play a pivotal role in this process [2,13,14,20,21]. However, the possibility of integration between AKT- and NF-κB-mediated signaling pathways during clasmatodendritic degeneration has not been reported.

Glutathione peroxidase-1 (GPx1) is the first identified selenoprotein that scavenges reactive oxygen species (ROS) through the reduction of H_2_O_2_ by using glutathione (GSH, an endogenous antioxidant) as a cofactor [22,23]. GPx1 regulates the induction of autophagy in response to ROS [24,25]. GPx1 downregulation is relevant to clasmatodendrosis in the hippocampus of chronic epilepsy rats, which is regulated by GSH biosynthesis. Briefly, GPx1 is significantly decreased in clasmatodendritic CA1 astrocytes, while it is increased in reactive CA1 astrocytes. N-acetylcysteine (NAC, a GSH precursor) restores GPx1 expression in clasmatodendritic astrocytes and alleviates clasmatodendrosis. In contrast, L-buthionine sulfoximine (BSO, an inducer of GSH depletion) aggravates clasmatodendrosis accompanied by GPx1 downregulation, independent of nuclear factor erythroid-2-related factor 2 (Nrf2) activity [15]. Since GPx1 plays an important role in astroglial viability against ROS-mediated toxicity [26,27], we have suggested that GSH-mediated GPx1 regulation may be related to clasmatodendrosis in CA1 astrocytes, although the regulatory signal pathways of these phenomena have not been fully explored.

As aforementioned, NAC induces GPx1 upregulation [15], which can inhibit NF-κB S536 phosphorylation [28,29]. In addition, GPx1 expression is transiently reduced in CA1 astrocytes at 3 day after status epilepticus (SE) when NF-κB transactivation is increased [15,30]. NAC also inhibits TNF-α-induced AKT activation and AKT-mediated NF-κB S536 phosphorylation [31]. Furthermore, GPx1 silencing drives the ROS-mediated AKT activation [32,33]. Considering these previous studies, it is likely that (1) NF-κB and GPx1 may reciprocally regulate each other and/or that (2) GPx1 may integrate between NF-κB- and AKT-mediated signaling pathways during clasmatodendritic degeneration, which have not been reported yet. Thus, we conducted the present study to elucidate these hypotheses.

Here, we demonstrate that NAC attenuated clasmatodendrosis by alleviating GPx1 downregulation, casein kinase 2 (CK2)-mediated NF-κB S529 and AKT-mediated NF-κB S536 phosphorylations. 2-[4,5,6,7-tetrabromo-2-(dimethylamino)-1H-benzo[d]imidazole-1-yl]acetic acid (TMCB; a selective CK2 inhibitor) relieved clasmatodendritic degeneration and GPx1 downregulation concomitant with the decreased NF-κB S529, S536 and AKT S473 phosphorylations. However, AKT inhibition by 3-chloroacetyl-indole (3CAI) did not affect GPx1 downregulation and CK2 tyrosine (Y) 255 and NF-κB S529 phosphorylations, although it mitigated clasmatodendrosis and NF-κB S536 phosphorylation. These unreported data suggest that seizure-induced oxidative stress may diminish GPx1 expression by increasing CK2-mediated NF-κB S529 phosphorylation, which would subsequently enhance NF-κB S536 phosphorylation by AKT hyperactivation, leading to autophagic astroglial degeneration.

## 2. Materials and Methods

### 2.1. Experimental Animals and Chemicals

Male Sprague Dawley (SD) rats (200–250 g) were cared under controlled environmental conditions (23–25 °C, 12 h light/dark cycle) and freely accessed to water and conventional rat diets. All experimental protocols described below were approved by the Institutional Animal Care and Use Committee of Hallym University (Hallym 2021-3, approval date: 17 May 2021). All reagents were obtained from Sigma-Aldrich (St. Louis, MO, USA), except as noted.

### 2.2. Chronic Epilepsy Rat Model

One day before pilocarpine treatment, rats were given LiCl (127 mg/kg, i.p.). The following day, animals were injected with atropine methylbromide (5 mg/kg i.p.) 20 min before pilocarpine (30 mg/kg, i.p.) treatment. To cease status epilepticus (SE), diazepam (Valium; Hoffmann-la Roche, Neuilly-sur-Seine, France; 10 mg/kg, i.p.) was administered 2 h after SE on-set and repeated, as needed. Control animals were given saline instead of pilocarpine. After SE induction, rats were monitored 8 h/day to identify chronic epilepsy activity [14,34].

### 2.3. NAC Treatment

Chronic epilepsy rats were given N-acetylcysteine (NAC, 70 mg/kg/day, i.p.) over a 7-day period [17]. Five hours after the last injection, the animals were used for experiments.

### 2.4. Infusion of TMCB and 3CAI

Animals were implanted with an infusion needle (Brain infusion kit 1, Alzet, Cupertino, CA, USA) into the right lateral ventricle (coordinates: 1 mm posterior; 1.5 mm lateral; 3.5 mm depth) under isoflurane anesthesia (3% induction, 1.5–2% for surgery, and 1.5% maintenance in a 65:35 mixture of N_2_O:O_2_), and connected with an Alzet 1007D osmotic pump (Alzet, Cupertino, CA, USA) containing (1) the vehicle, (2) TMCB (0.5 μM) or (3) 3CAI (25 μM). Seven days after surgery, the animals were used for experiments [14,30].

### 2.5. Western Blot

Under urethane anesthesia (1.5 g/kg, i.p.), rats were decapitated, and the hippocampus was rapidly dissected out and homogenized in lysis buffer containing protease inhibitor cocktail (Roche Applied Sciences, Branford, CT, USA) and phosphatase inhibitor cocktail (PhosSTOP^®^, Roche Applied Science, Branford, CT, USA). The protein concentration was measured using a Micro BCA Protein Assay Kit (Pierce Chemical, Dallas, TX, USA). Thereafter, Western blotting was performed by the standard protocol (*n* = 7 rats in each group). After electrophoresis, proteins were transferred to polyvinylidene fluoride membranes that were subsequently incubated with a blocking solution followed by immunoblotting with the primary antibody (Table 1). For chemiluminescent detection and analysis, an ImageQuant LAS4000 system (GE Healthcare Korea, Seoul, South Korea) was used. The β-actin value was used for the normalization of each protein value. The phosphoprotein/total protein ratio was represented as the phosphorylation ratio [14,30,34].

### 2.6. Tissue Preparation and Immunohistochemistry

Animals were anesthetized with urethane anesthesia (1.5 g/kg, i.p.) and perfused with 4% paraformaldehyde in 0.1 M phosphate buffer (PB, pH 7.4) through the left ventricle followed by post-fixation in the same fixative overnight. After immersion with 30% sucrose overnight, brains were sectioned at 30 μm. Sections were blocked with 3% bovine serum albumin in PBS for 30 min, and later incubated overnight with mixtures of primary antibodies (Table 1) in PBS containing 0.3% Triton X-100. After washing, tissues were reacted with Brilliant Violet-421, Cy2- or Cy3-fluorescent dye conjugated secondary antibodies (Jackson Immuno Research Laboratories, West Grove, PA, USA). The fluorescent intensity was quantified in the randomly selected 2–3 reactive astrocytes or clasmatodendritic astrocytes in the stratum radiatum of the CA1 region (*n* = 7 rats in each group) with AxioVision Rel. 4.8 (Carl Zeiss Korea, Seoul, Republic of Korea) and ImageJ software. For quantification of clasmatodendritc astrocytes, cell counts were conducted in areas of interest (1 × 10^4^ μm^2^) of 10 sections per each animal [14,34].

### 2.7. Data Analysis

The Mann–Whitney test was applied to analyze statistical significance of data obtained from two groups. The Kruskal–Wallis test with Dunn–Bonferroni post hoc test was used for the comparison of data obtained four groups. The Spearman test was applied to identify the relationship between two variables. A *p*-value less than 0.05 was considered significant.

## 3. Results

### 3.1. NAC Restores GPx1 Expression and Inhibits CK2-Mediated NF-κB S529 Phosphorylation in Clasmatodendritic CA1 Astrocytes

NF-κB signaling pathway activates autophagy. In particular, NF-κB S529 phosphorylation is involved in clasmatodendritic astrocytes [2,35]. Since NF-κB at S529 site is phosphorylated by CK2 [36], we investigated the effects of NAC on GPx1 expression and CK2-mediated NF-κB S529 phosphorylation in clasmatodendritic astrocytes.

**Figure 1:**
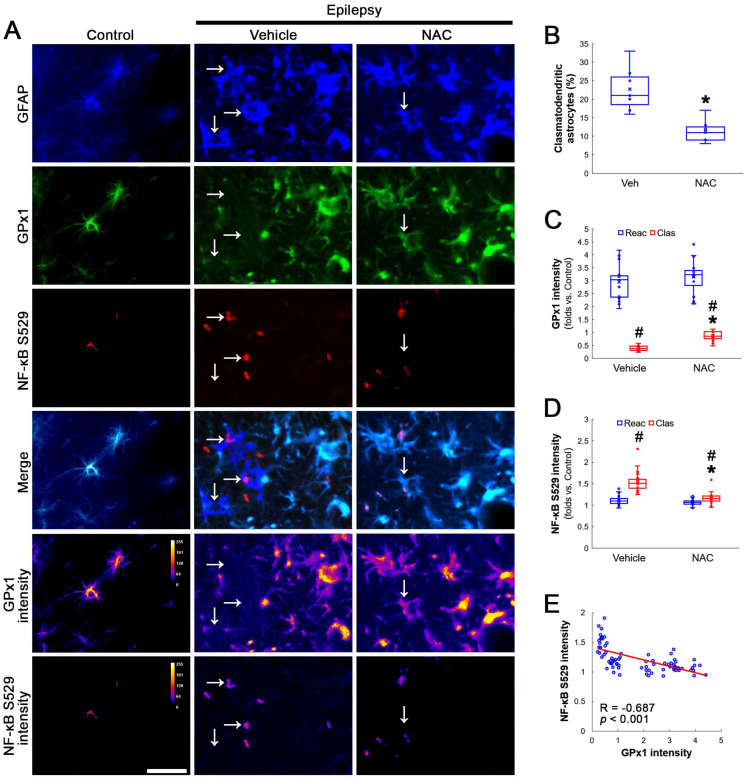
**Effects of NAC on GPx1 expression and NF-κB S529 phosphorylation in CA1 astrocytes.** Compared to control rats, GPx1 expression is increased in reactive CA1 astrocytes (Reac), while it is reduced in clasmatodendritic (vacuolized) CA1 astrocytes (Clas, arrows). However, the NF-κB S529 signal is enhanced only in clasmatodendritic CA1 astrocytes. CK2 Y255 phosphorylation is diminished in the whole hippocampus of chronic epilepsy rats. Compared to the vehicle (Veh), NAC ameliorates GPx1 downregulation and NF-κB S529 phosphorylation in clasmatodendritic astrocytes, accompanied by the reduced CK2 Y255 phosphorylation. (**A**) Representative photos of GPx1 expression, NF-κB S529 signal and their intensities. Bar = 25 μm. (**B**) Quantification of clasmatodendritic degeneration in CA1 astrocytes (* *p* < 0.05 vs. vehicle, *n* = 7 rats, respectively; Mann–Whitney test). (**C**,**D**) Quantification of GPx1 and NF-κB S529 intensities in CA1 astrocytes (*^,#^ *p* < 0.05 vs. vehicle and reactive astrocytes, respectively, *n* = 20 cells in 7 rats, respectively; Kruskal–Wallis test with Dunn–Bonferroni post hoc test). (**E**) Linear regression analysis between GPx1 and NF-κB S529 intensities in reactive and clasmatodendritic CA1 astrocytes of chronic epilepsy rats (*n* = 80 cells in 14 rats; Spearman test).

Compatible with a previous study [16], NAC ameliorated clasmatodendritic degeneration of CA1 astrocytes (Figure 1A,B). Clasmatodendritic CA1 astrocytes showed GPx1 downregulation, although reactive CA1 astrocytes exhibited GPx1 upregulation (Figure 1A,C). Compared to the vehicle, NAC increased GPx1 expression in clasmatodendritic (vacuolized) astrocytes, but not in reactive astrocytes (Figure 1A,C). In contrast to GPx1, NF-κB S529 phosphorylation was enhanced in clasmatodendritic astrocytes, as compared reactive astrocytes (Figure 1A,D). NAC abolished NF-κB S529 phosphorylation in clasmatodendritic CA1 astrocytes, but not in reactive astrocytes (Figure 1A,D). Thus, NF-κB S529 phosphorylation showed an inverse correlation with GPx1 expression (Figure 1E).

**Figure 2:**
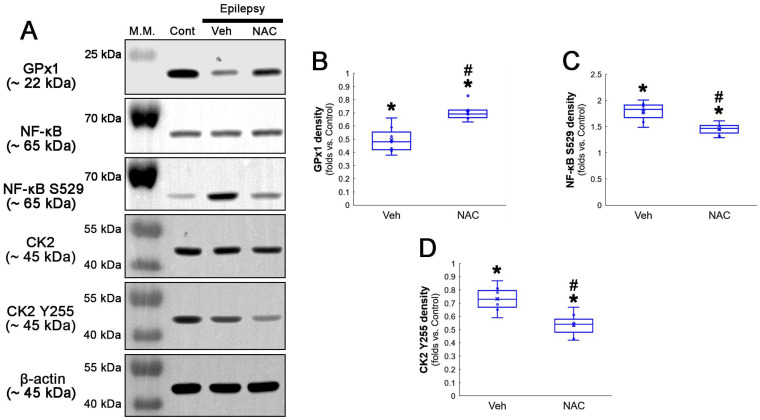
**Western blot data representing the effects of NAC on GPx1 expression, NF-κB S529, and CK2 Y255 phosphorylations.** Consistent with the immunofluorescent study (Figure 1), NAC increases GPx1 expression, but diminishes NF-κB S529 phosphorylation level, as compared to the vehicle (Veh). In addition, CK2 Y255 phosphorylation is decreased in the whole hippocampus of chronic epilepsy rats, which is further reduced by NAC. (**A**) Representative Western blot of GPx1, NF-κB, NF-κB S529, CK2 and CK2 Y255 levels. (**B**–**D**) Quantification of GPx1, NF-κB S529 and CK2 Y255 phosphorylation levels based on Western blot data (*^,#^ *p* < 0.05 vs. control rats and vehicle-treated epilepsy rats, *n* = 7 rats, respectively; Kruskal–Wallis test with Dunn–Bonferroni post hoc test).

Compatible with immunofluorescent studies, Western blot data also revealed that NAC augmented GPx1 expression, but reduced NF-κB S529 phosphorylation level, as compared to the vehicle (Figure 2A–C and Figure S1). Since NF-κB S529 phosphorylation is regulated by CK2, which increases NF-κB-mediated nuclear transcriptional activity [36], we further evaluated the effect of NAC on CK2 phosphorylation (activity). Western blot data revealed that CK2 Y255 phosphorylation was reduced in the hippocampus of chronic epilepsy rats, as compared to control animals (Figure 2A,D and Figure S1). Compared to the vehicle, NAC further diminished CK2 Y255 phosphorylation without altering its total protein level (Figure 2A,D and Figure S1). Considering that inhibition of the Src/CK2 signaling pathway is one of the insufficient adaptive responses to seizures [14,37,38], our findings indicated that CK2-mediated NF-κB S529 phosphorylation may lead to clasmatodendrosis, accompanied by GPx1 downregulation.

### 3.2. NAC Diminished AKT-Mediated NF-κB S536 Phosphorylation in Clasmatodendritic CA1 Astrocytes

**Figure 3:**
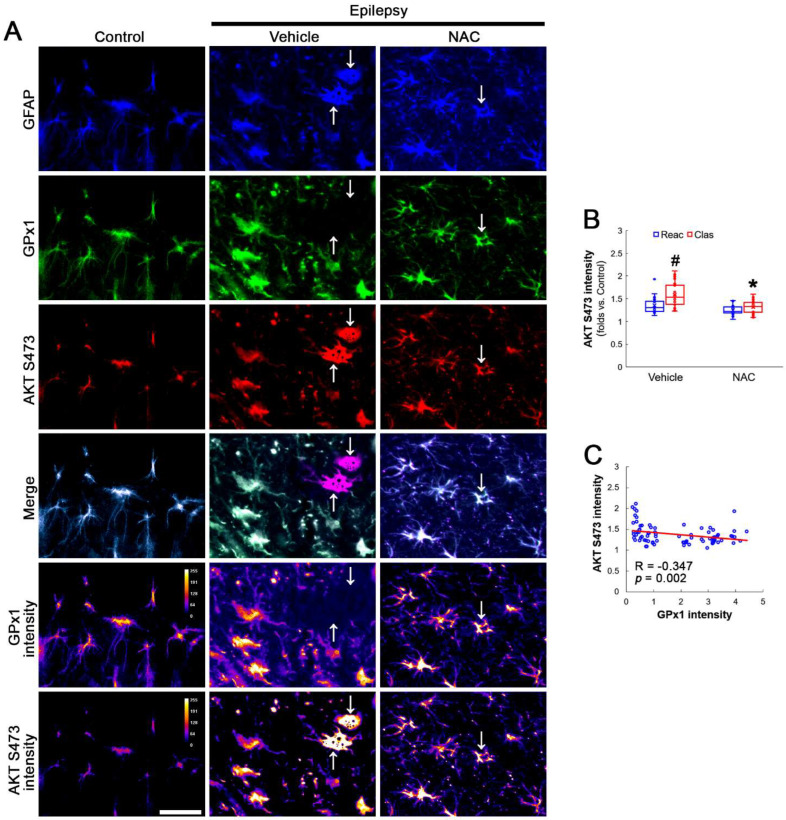
**Effects of NAC on GPx1 expression and AKT S473 phosphorylation in CA1 astrocytes.** Compared to control rats, AKT S473 phosphorylation is enhanced in clasmatodendritic (vacuolized) CA1 astrocytes (Clas, arrows) more than reactive CA1 astrocytes (Reac), which is attenuated by NAC treatment. (**A**) Representative photos of GPx1 expression, AKT S473 signal and their intensities. Bar = 25 μm. (**B**) Quantification of AKT S473 intensity in CA1 astrocytes (*^,#^ *p* < 0.05 vs. vehicle and reactive astrocytes, respectively, *n* = 20 cells in 7 rats, respectively; Kruskal–Wallis test with Dunn–Bonferroni post hoc test). (**C**) Linear regression analysis between GPx1 and AKT S473 intensities in reactive and clasmatodendritic CA1 astrocytes of chronic epilepsy rats (*n* = 80 cells in 14 rats; Spearman test).

**Figure 4:**
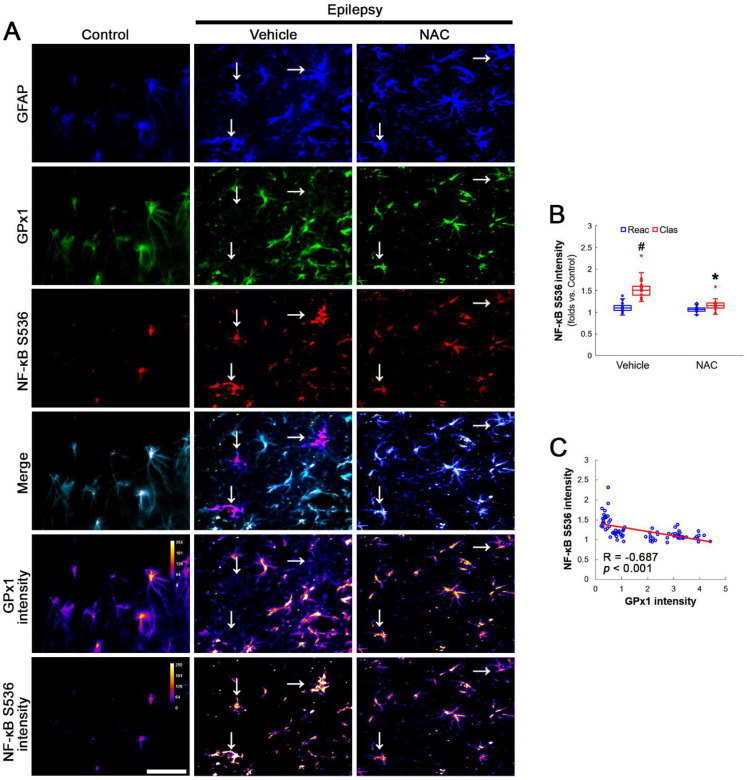
**Effects of NAC on GPx1 expression and NF-κB S536 phosphorylation in CA1 astrocytes.** Compared to control rats, NF-κB S536 signal is increased in clasmatodendritic (vacuolized) CA1 astrocytes (Clas, arrows), but not reactive CA1 astrocytes (Reac), which is attenuated by NAC treatment. (**A**) Representative photos of GPx1 expression, NF-κB S536 signal and their intensities. Bar = 25 μm. (**B**) Quantification of NF-κB S536 intensity in CA1 astrocytes (*^,#^ *p* < 0.05 vs. vehicle and reactive astrocytes, respectively, *n* = 20 cells in 7 rats, respectively; Kruskal–Wallis test with Dunn–Bonferroni post hoc test). (**C**) Linear regression analysis between GPx1 and NF-κB S536 intensities in reactive and clasmatodendritic CA1 astrocytes of chronic epilepsy rats (*n* = 80 cells in 14 rats; Spearman test).

AKT S473 hyperphosphorylation causes bax-interacting factor 1 (Bif-1)-mediated astroglial autophagy [13,14]. AKT activation also stimulates NF-κB S536 phosphorylation [39]. Interestingly, deletion or inhibition of GPx1 increases NF-κB S536 phosphorylation [28,29] and NFκB S536 phosphorylation is critical for autophagy in response to oxidative stress [40,41]. Furthermore, NAC inhibits TNF-α-induced AKT S473 and NF-κB S536 phosphorylation [31]. Thus, we explored whether NF-κB S536 phosphorylation is involved in AKT-mediated clasmatodendrosis and NAC abolishes this pathway. Compared to intact astrocytes, both reactive astrocytes and clasmatodendritic CA1 astrocytes showed AKT S473 hyperphosphorylation (Figure 3A,B). However, AKT S473 intensity in clasmatodendritic astrocytes was higher than that in reactive astrocytes (Figure 3A,B). NAC attenuated AKT S473 hyperphosphorylation in clasmatodendritic astrocytes, but not in reactive astrocytes (Figure 3A,B). AKT S473 phosphorylation showed an inverse proportion with GPx1 expression (Figure 3C). Similar to the case of AKT S473 phosphorylation, NF-κB S536 phosphorylation was enhanced in clasmatodendritic astrocytes, compared to reactive astrocytes (Figure 4A,B). NAC abolished NF-κB S536 phosphorylation in clasmatodendritic CA1 astrocytes, but not in reactive astrocytes (Figure 4A,B). Linear regression analysis showed an inverse proportional relationship between GPx1 and NF-κB S536 phosphorylation (Figure 4C).

**Figure 5:**
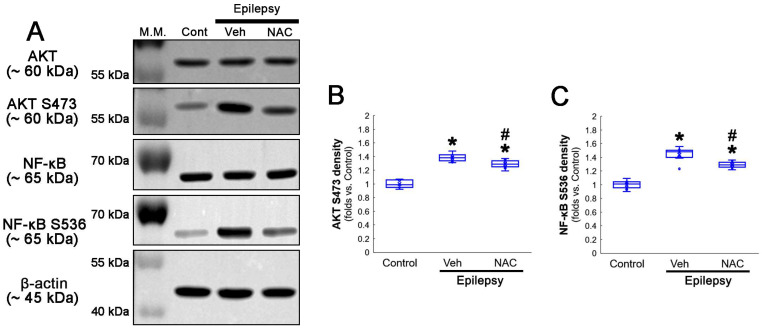
**Western blot data representing the effects of NAC on AKT S473 and NF-κB S536 phosphorylations.** Consistent with immunofluorescent study (Figure 3 and Figure 4), NAC diminishes AKT S473 and NF-κB S536 phosphorylation levels, as compared to the vehicle (Veh). (**A**) Representative Western blot of AKT, AKT S473, NF-κB and NF-κB S536 levels. (**B**,**C**) Quantification of AKT S473 and NF-κB S536 phosphorylation levels based on Western blot data (*^,#^ *p* < 0.05 vs. control animals and vehicle-treated epilepsy rats, respectively, *n* = 7 rats, respectively; Kruskal–Wallis test with Dunn–Bonferroni post hoc test).

Compatible with immunofluorescent studies, Western blot data also revealed that NAC augmented AKT S473 and NF-κB S536 phosphorylation levels, as compared to the vehicle (Figure 5A–C and Figure S2). These findings indicate that AKT-mediated NF-κB S536 phosphorylation may participate in clasmatodendritic degeneration, and that NAC may ameliorate clasmatodendrosis by inhibiting this pathway as well as CK2-mediated NF-κB S529 phosphorylation.

### 3.3. CK2 Inhibition Restores GPx1 Upregulation and Attenuates NF-κB and AKT Phosphorylations in Clasmatodendritic CA1 Astrocytes

**Figure 6:**
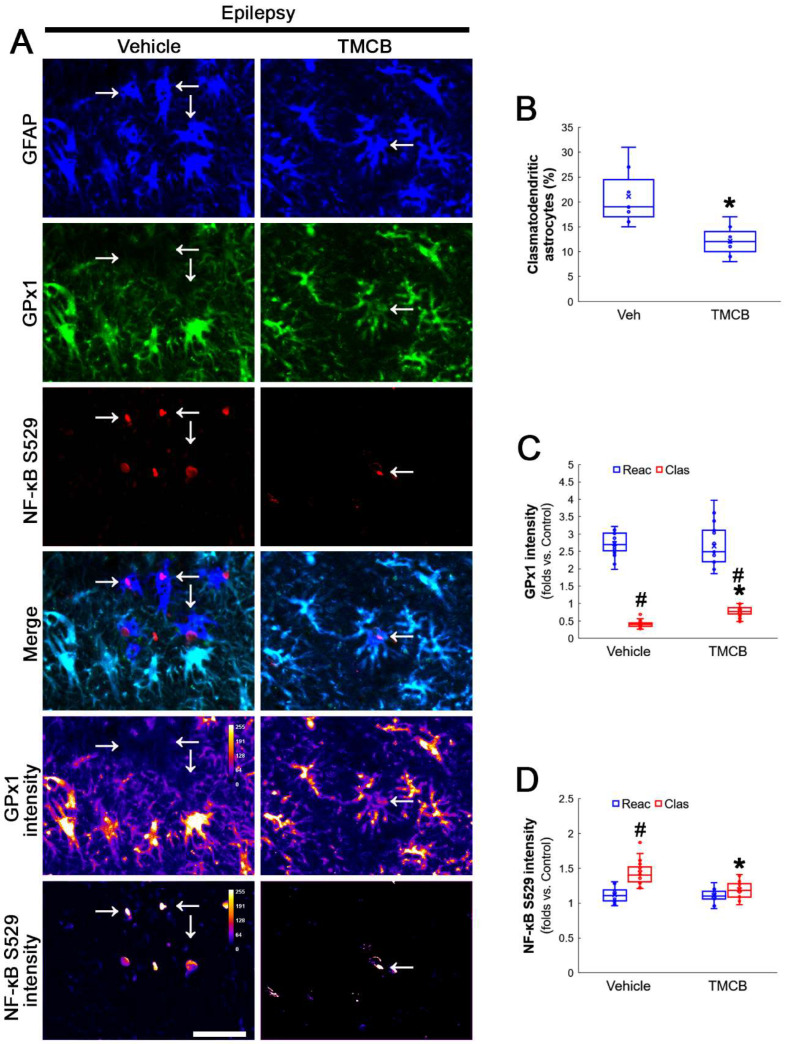
**Effects of TMCB on GPx1 expression and NF-κB S529 phosphorylation in CA1 astrocytes.** Compared to the vehicle, TMCB attenuates clasmatodendritic degeneration concomitant with the enhanced GPx1 expression and the decreased NF-κB S529 phosphorylation in clasmatodendritic (vacuolized) CA1 astrocytes (Clas, arrows), but not reactive CA1 astrocytes (Reac). (**A**) Representative photos of GPx1 expression, NF-κB S529 signal and their intensities. Bar = 25 μm. (**B**) Quantification of clasmatodendritic degeneration in CA1 astrocytes (* *p* < 0.05 vs. vehicle, *n* = 7 rats, respectively; Mann–Whitney test). (**C**,**D**) Quantification of GPx1 and NF-κB S529 intensities in CA1 astrocytes (*^,#^ *p* < 0.05 vs. vehicle and reactive astrocytes, respectively, *n* = 20 cells in 7 rats, respectively; Kruskal–Wallis test with Dunn–Bonferroni post hoc test).

**Figure 7:**
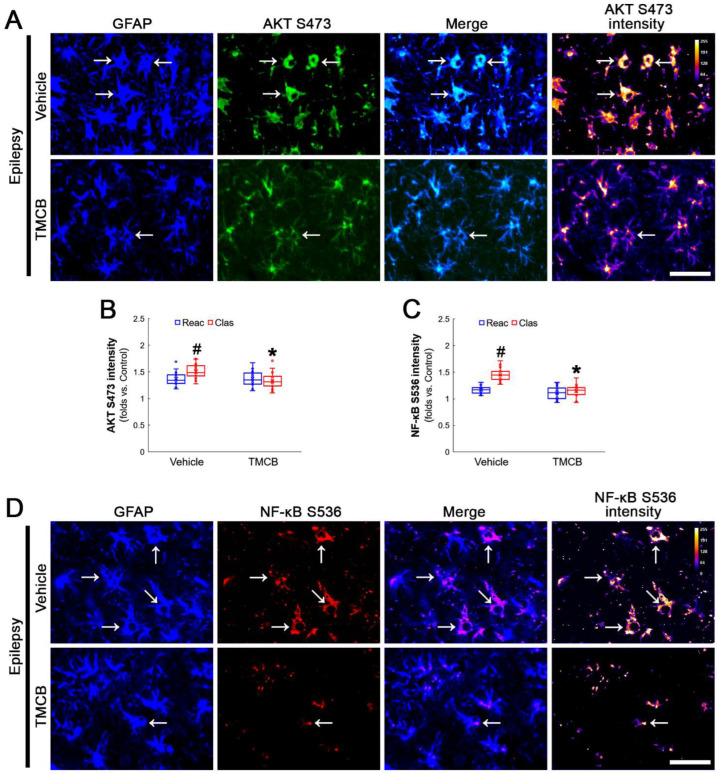
**Effects of TMCB on AKT S473 and NF-κB S536 phosphorylations in CA1 astrocytes.** Compared to the vehicle, TMCB ameliorates NF-κB S536, but not AKT S473, phosphorylation in clasmatodendritic (vacuolized) CA1 astrocytes (Clas, arrows), but not reactive CA1 astrocytes (Reac). (**A**) Representative photos of AKT S473 phosphorylation and its intensities. Bar = 25 μm. (**B**,**C**) Quantification of AKT S473 and NF-κB S536 intensity in CA1 astrocytes (*^,#^ *p* < 0.05 vs. vehicle and reactive astrocytes, respectively, *n* = 20 cells in 7 rats, respectively; Kruskal–Wallis test with Dunn–Bonferroni post hoc test). (**D**) Representative photos of NF-κB S536 phosphorylation and its intensities. Bar = 25 μm.

Next, we applied TMCB (a selective CK2 inhibitor) to identify whether the CK2 signaling pathway would induce GPx1 downregulation during clasmatodendritic degeneration. Similar to the case of NAC, TMCB attenuated clasmatodendritic degeneration of CA1 astrocytes (Figure 6A,B). Compared to the vehicle, TMCB increased GPx1 expression in clasmatodendritic astrocytes, but not in reactive astrocytes (Figure 6A,C). TMCB abolished NF-κB S529 phosphorylation in clasmatodendritic CA1 astrocytes, but not in reactive astrocytes (Figure 6A,D). Furthermore, TMCB abrogated AKT S473 hyperphosphorylation in clasmatodendritic astrocytes, but not in reactive astrocytes (Figure 7A,B). TMCB also diminished NF-κB S536 phosphorylation in clasmatodendritic CA1 astrocytes, but not in reactive astrocytes (Figure 7C,D).

**Figure 8:**
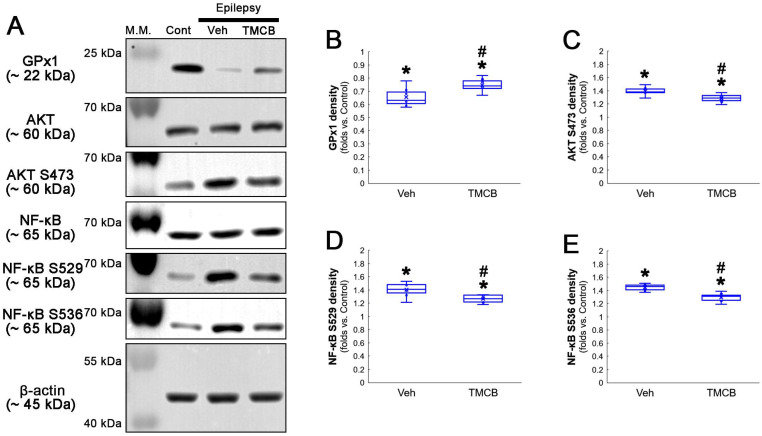
**Western blot data representing the effects of TMCB on GPx1 expression, AKT S473, NF-κB S529 and NF-κB S536 phosphorylations**. Consistent with immunofluorescent study (Figure 6 and Figure 7), TMCB increases GPx1 expression, but reduces AKT S473, NF-κB S529 and NF-κB S536 phosphorylation levels, as compared to the vehicle (Veh). (**A**) Representative Western blot of GPx1, AKT, AKT S473, NF-κB, NF-κB S529 and NF-κB S536 levels. (**B**–**E**) Quantification of GPx1 expression, AKT S473, NF-κB S529 and NF-κB S536 phosphorylation levels based on Western blot data (*,^#^ *p* < 0.05 vs. control rats and vehicle-treated epilepsy rats, respectively, *n* = 7 rats, respectively; Kruskal–Wallis test with Dunn–Bonferroni post hoc test).

Western blot data also demonstrated that TMCB increased GPx1 expression, but decreased AKT S473, NF-κB S529 and NF-κB S536 phosphorylation levels, as compared to the vehicle (Figure 8A–E and Figure S3). These findings indicate that CK2-mediated NF-κB S529 phosphorylation may diminish GPx1 expression during clasmatodendrosis, and that AKT-mediated NF-κB S536 phosphorylation may be a consequence of GPx1 downregulation induced by this pathway.

### 3.4. AKT Inhibition Attenuates Clasmatodendrosis and NF-κB S536 Phosphorylation without Affecting GPx1 Level and CK2-Mediated NF-κB S529 Phosphorylation in Clasmatodendritic CA1 Astrocytes

**Figure 9:**
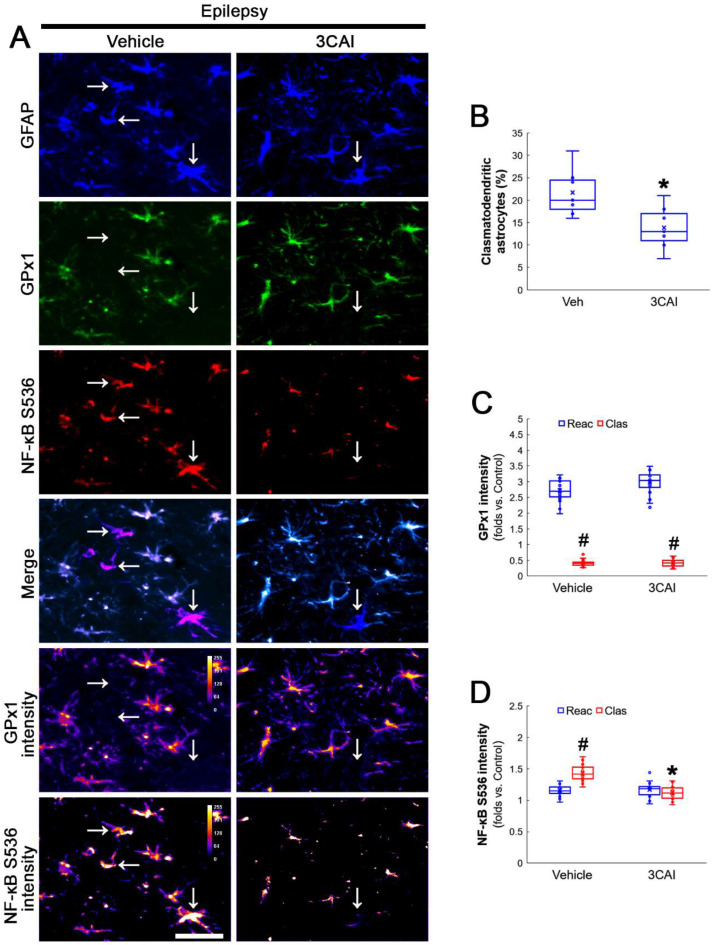
**Effects of 3CAI on GPx1 expression and NF-κB S536 phosphorylation in CA1 astrocytes.** Compared to the vehicle, 3CAI attenuates clasmatodendritic degeneration concomitant and the increased NF-κB S536 phosphorylation in clasmatodendritic (vacuolized) CA1 astrocytes (Clas, arrows), but not reactive CA1 astrocytes (Reac), while it does not affect GPx1 expression level. (**A**) Representative photos of GPx1 expression and NF-κB S536 signal and their intensities. Bar = 25 μm. (**B**) Quantification of clasmatodendritic degeneration in CA1 astrocytes (* *p* < 0.05 vs. vehicle, *n* = 7 rats, respectively; Mann–Whitney test). (**C**,**D**) Quantification of GPx1 and NF-κB S536 intensities in CA1 astrocytes (*^,#^ *p* < 0.05 vs. vehicle and reactive astrocytes, respectively, *n* = 20 cells in 7 rats, respectively; Kruskal–Wallis test with Dunn–Bonferroni post hoc test).

**Figure 10:**
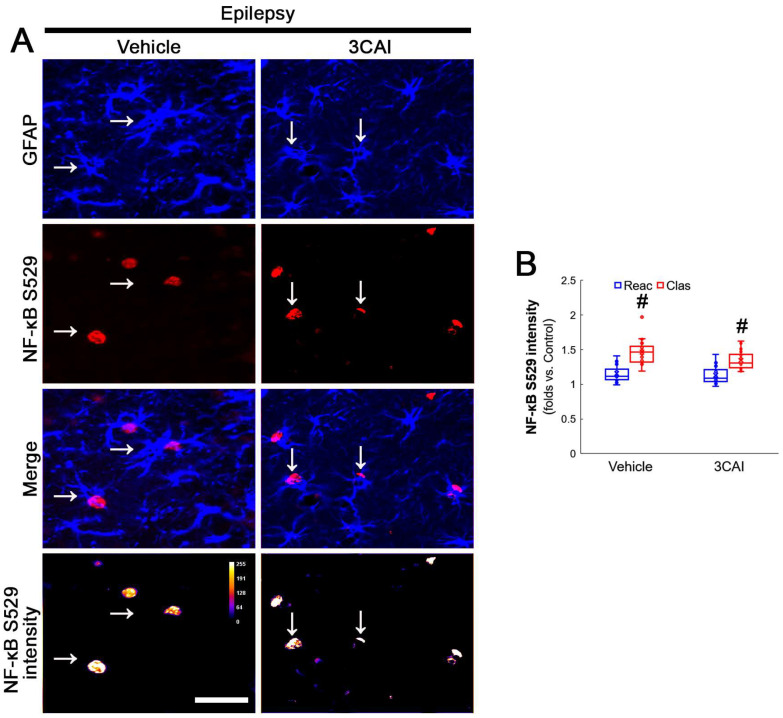
**Effects of 3CAI on NF-κB S529 and CK2 Y255 phosphorylations in CA1 astrocytes.** Compared to the vehicle, 3CAI does not influence NF-κB S529 in clasmatodendritic (vacuolized) CA1 astrocytes (Clas, arrows) and reactive CA1 astrocytes (Reac). CK2 Y255 phosphorylation in the whole hippocampus is also unaffected by 3CAI treatment. (**A**) Representative photos of the NF-κB S529 signal and its intensities. Bar = 25 μm. (**B**) Quantification of NF-κB S529 intensity in CA1 astrocytes (^#^ *p* < 0.05 vs. reactive astrocytes, *n* = 20 cells in 7 rats, respectively; Kruskal–Wallis test with Dunn–Bonferroni post hoc test).

To confirm the role of AKT-mediated NF-κB S536 phosphorylation in clasmatodendritic degeneration, we applied 3CAI to chronic epilepsy rats. 3CAI ameliorated clasmatodendritic degeneration of CA1 astrocytes (Figure 9A,B). 3CAI also decreased NF-κB S536 phosphorylation in clasmatodendritic astrocytes, but not in reactive astrocytes (Figure 9A,C). However, 3CAI could not affect reduced GPx1 expression in clasmatodendritic astrocytes (Figure 9A,D). In addition, 3CAI did not influence increased NF-κB S529 phosphorylation in CA1 astrocytes (Figure 10A,B).

**Figure 11:**
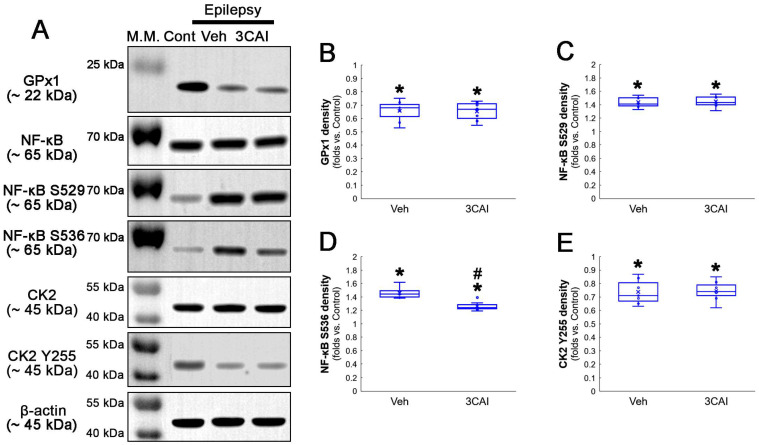
**Western blot data representing the effects of 3CAI on GPx1 expression, NF-κB S529, NF-κB S536 and CK2 Y255 phosphorylations**. Consistent with the immunofluorescent study (Figure 9 and Figure 10), 3CAI reduces only the NF-κB S536 phosphorylation level without affecting GPx1 expression, NF-κB S529 and CK2 Y255 phosphorylations, as compared to the vehicle (Veh). (**A**) Representative Western blot of GPx1, NF-κB, NF-κB S529, NF-κB S536, CK2 and CK2 Y255 levels. (**B**–**E**) Quantification of GPx1 expression, AKT S473, NF-κB S529 and NF-κB S536 phosphorylation levels based on Western blot data (*^,#^ *p* < 0.05 vs. control rats and vehicle-treated epilepsy rat, respectively, *n* = 7 rats, respectively; Kruskal–Wallis test with Dunn–Bonferroni post hoc test).

Western blot data revealed that 3CA1 reduced the NF-κB S536 phosphorylation level without affecting GPx1 expression, NF-κB S529 and CK2 Y255 phosphorylation (Figure 11A–E and Figure S4). Regarding the GPx1-mediated inhibition of NF-κB S536 phosphorylation [25,26], these findings indicate that the CK2-NF-κB S529-GPx1 signaling pathway may be an upstream regulator of AKT-mediated NF-κB S536 phosphorylation during clasmatodendritic degeneration.

## 4. Discussion

Astroglial activation generates H_2_O_2_ that evokes an imbalance of redox homeostasis in the brain [42]. Therefore, the defense system removing H_2_O_2_ is essential for astroglial viability. GPx1 plays an important role in GSH-mediated H_2_O_2_ elimination [22,23]. Indeed, GPx expression is increased in glial cells around surviving neurons [43] and GPx1 inhibits the ROS-mediated AKT activation [32,33]. In the present study, GPx1 was upregulated in reactive CA1 astrocytes, suggesting that increased GPx1 expression in reactive astrocytes may be an adaptive response against oxidative stress. However, GPx1 expression was significantly diminished in clasmatodendritic CA1 astrocytes concomitant with increased NF-κB S529 phosphorylation, which was recovered by NAC. NF-κB signaling pathway activates autophagy after heat shock [35]. Indeed, NF-κB S529, but not S276 and S311, phosphorylation is involved in clasmatodendritic astrocytes [2]. Since NAC acts as a direct ROS scavenger *per se* as well as a GSH precursor leading to increased GPx1/2 expression [44,45,46], our findings suggest that the antioxidant properties of NAC may improve GPx1 downregulation in clasmatodendritic astrocytes by inhibiting NF-κB S529 phosphorylation.

S529 phosphorylation increases NF-κB-mediated nuclear transcriptional activity, which is regulated by CK2 [36]. CK2 is a highly conserved and constitutively active serine/threonine kinase that promotes cell viability, proliferation and differentiation [47,48]. CK2 activity is enhanced by phosphorylation of Y255 and T360/S362 sites, which are modulated by the Src family and extracellular signal-regulated kinase 1/2 (ERK1/2), respectively [49,50]. In the epileptic hippocampus, CK2 Y255, but not T360/S362, phosphorylation is decreased as an insufficient and maladaptive response to inactivation/downregulation of phosphatase and tensin homolog deleted on chromosome 10 (PTEN). Furthermore, inhibition of Src-mediated CK2 Y255 phosphorylation further ameliorates PTEN downregulation/phosphorylation and clasmatodendrosis [14,38]. Furthermore, Src inhibition enhances GPx1 levels [51] and Src kinase upregulation inhibits GPx1 activity [52]. Most of all, NF-κB activation increases proinflammatory cytokines, including TNF-α, which abrogates the compensatory GPx1 induction following oxidative stress [27,53]. Indeed, NAC suppresses ROS-mediated NF-κB and subsequent mRNA expression of chemokines in human astrocytes [54] and fully blocks ROS-induced CK2 upregulation that induces NF-κB activation [55,56]. Compatible with these reports, the present data show that CK2 inhibition by NAC and TMBC effectively enhanced GPx1 expression in vacuolized CA1 astrocytes concomitant with reduced NF-κB S529 phosphorylation. Therefore, our findings indicate that CK2-mediated NF-κB S529 phosphorylation may be an upstream pathway of GPx1 downregulation.

The present data show AKT S473 hyperphosphorylation in clasmatodendritic CA1 astrocytes exhibiting low GPx1 intensity. Oxidative stress triggers AKT activation [57], which inhibits ROS-induced GPx1 upregulation [58]. Therefore, the present data are simply interpreted as that AKT may be one of the upstream molecules to suppress GPx1 expression in clasmatodendritic astrocytes. In the present study, however, AKT inhibition by 3CAI did not improve GPx1 downregulation in clasmatodendritic astrocytes, although it attenuated clasmatodendrosis. Therefore, our findings indicate that AKT S473 hyperphosphorylation may not be relevant to reduced GPx1 expression during clasmatodendritic degeneration.

On the other hand, CK2 also activates AKT by phosphorylation at S129 site [59,60,61]. In addition, the present study reveals that both CK2 inhibition by TMCB and AKT inhibition by 3CAI attenuated clasmatodendritic degeneration. Considering these, it is plausible that CK2-mediated AKT S129 phosphorylation would also elicit clasmatodendrosis by NF-κB S536 phosphorylation. However, CK2-mediated AKT S129 phosphorylation is necessary for the cell viability in HEK-293T cells [59]. Indeed, CX-4945 (a CK2 inhibitor) exerts strong anti-proliferative activity by blocking AKT S129 phosphorylation in cancer cells [60,61]. Therefore, it is likely that CK2-mediated AKT S129 phosphorylation may not be involved in clasmatodendritic degeneration or astroglial viability in the epileptic hippocampus.

The decreased GPx1 expression also elicits the activation of the redox-sensitive NF-κB canonical pathway and increases autophagic flux [62]. Indeed, GPx1 deletion increases NF-κB S536 phosphorylation [28], which is critical for autophagy in response to oxidative stress [40,41]. Consistent with a previous study demonstrating NAC-induced AKT and NF-κB inhibition [31], the present study demonstrates that NF-κB S536 phosphorylation was also enhanced in clasmatodendritic CA1 astrocytes showing AKT S473 hyperphosphorylation, which were attenuated by NAC and TMCB. However, AKT inhibition by 3CAI did not affect the reduced GPx1 level and the enhanced NF-κB S529 phosphorylation in clasmatodendritic astrocytes, although it attenuated clasmatodendrosis and NF-κB S536 phosphorylation. Considering AKT-mediated NF-κB S536 phosphorylation [39,63], our findings indicate that AKT-mediated NF-κB S536 phosphorylation may be also involved in clasmatodendritic degeneration, accompanied by the AKT/GSK-3β/Bif-1 signaling pathway. Since GPx1 inhibits NF-κB S536 phosphorylation [29] and CK2 inhibition diminishes AKT S473 phosphorylation [64,65], the present data also suggest that the enhanced AKT-mediated S536 phosphorylation may be a consequence from CK2-NF-κB S529-mediated GPx1 downregulation. Therefore, it is likely that that antioxidative capacity of NAC may be attributed to GPx1 upregulation by inhibiting CK2-NF-κB S529-mediated signaling pathway in clasmatodendritic astrocytes, independent of the AKT-NF-κB S536-mediated signaling pathway.

Astrocytes contribute to the slow afterhyperpolarizing potential (sAHP), which is a major intrinsic mechanism of neuronal inhibition and its termination [66]. 4,5,6,7-Tetrabromotriazole (TBB, a CK2 inhibitor) augments sAHP [67]. Since the inhibition of clasmatodendrosis shortens seizure duration in chronic epilepsy rats [14], the present data provide evidence that clasmatodendrosis may be an epiphenomenon maintaining prolonged seizure duration in the epileptic hippocampus.

## 5. Conclusions

**Figure 12:**
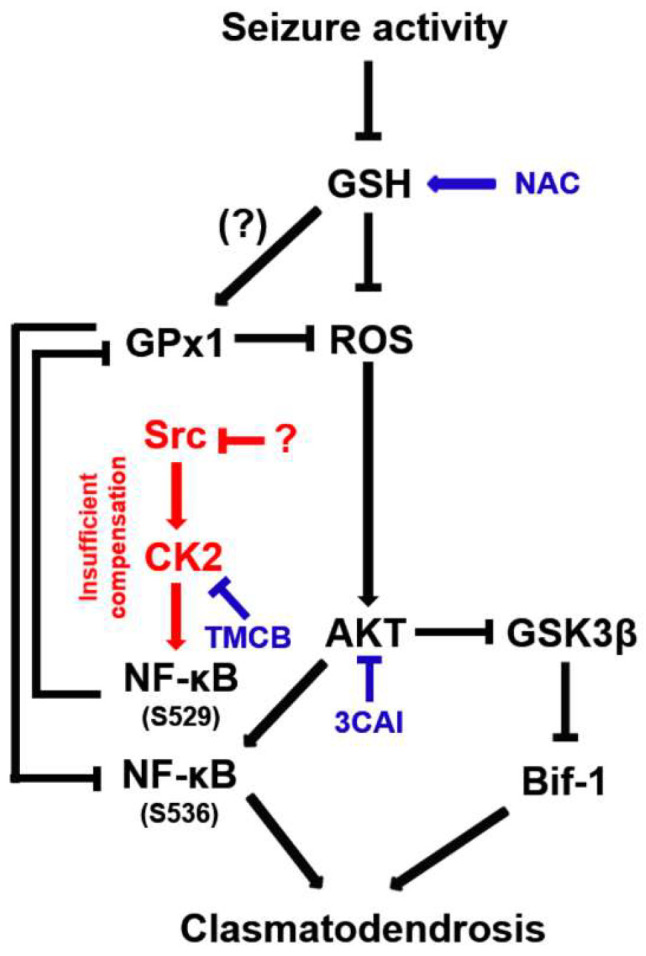
Schematic depiction representing the distinct role of NF-κB phosphorylation in clasmatodendritic CA1 astrocytes based on the present data and previous reports. Seizure activity decreases the GSH level and subsequently increases the ROS level. Aberrant CK2-mediated NF-κB S529 phosphorylation participates in GPx1 downregulation, which abolishes the GPx1-mediated inhibition of NF-κB S536 phosphorylation induced by AKT hyperactivation. In turn, the enhanced NF-κB S536 phosphorylation is involved in clasmatodendritic degeneration concomitant with AKT-mediated Bif-1 activation.

The present study demonstrates for the first time that CK2-mediated NF-κB S529 phosphorylation evoked GPx1 downregulation in clasmatodendritic astrocytes, which subsequently led to AKT-mediated NF-κB S536 phosphorylation facilitating this autophagic astroglial degeneration (Figure 12). Therefore, our findings suggest that GPx1 may integrate between CK2- and AKT-mediated signaling pathways during clasmatodendrosis induced by oxidative stress.

## Figures and Tables

**Table 1:** Primary antibodies used in the present study.

Antigen	Host	Manufacturer (Catalog Number)	Dilution Used
AKT	Rabbit	Cell signaling (#9272)	1:1000 (WB)
CK2	Mouse	Millipore (#05-1431)	1:1000 (WB)
GFAP	Mouse	Millipore (#MAB3402)	1:2000 (IH)
GPx1	Sheep	Biosensis (#S-072-100)	1:2000 (IH) 1:10,000 (WB)
NF-κB	Rabbit	Abcam (#ab16502)	1:500 (IH) 1:2000 (WB)
NF-κB S529	Rabbit	Abcam (#ab47395)	1:100 (IH) 1:1000 (WB)
NF-κB S536	Rabbit	Abcam (#ab28856)	1:100 (IH) 1:1000 (WB)
p-AKT S473	Rabbit	Cell signaling (#4060)	1:250 (IH) 1:1000 (WB)
p-CK2 Y255	Rabbit	Invitrogen (#PA5-38831)	1:1000 (WB)
β-actin	Mouse	Sigma (#A5316)	1:5000 (WB)

IH: Immunohistochemistry; WB: Western blot.

## Data Availability

Data sharing is not applicable to this article.

## Supplementary Materials

The following supporting information can be downloaded at: https://www.mdpi.com/article/10.3390/antiox12051020/s1. Figure S1: Full-length images of Western blots in Figure 2A. Figure S2: Full-length images of Western blots in Figure 5A. Figure S3: Full-length images of Western blots in Figure 8A. Figure S4: Full-length images of Western blots in Figure 11A.
