# Supplementary material for: Distinct Roles of CK2- and AKT-Mediated NF-κB Phosphorylations in Clasmatodendrosis (Autophagic Astroglial Death) within the Hippocampus of Chronic Epilepsy Rats
Source: Antioxidants (Basel). 2023 Apr 28;12(5):1020. doi: 10.3390/antiox12051020 (PMC10215642; doi:10.3390/antiox12051020)
Supplement: Supplementary file 1 [file antioxidants-12-01020-s001.zip › antioxidants-2336994-supplementary.pdf]

Distinct roles of CK2 and AKT-mediated NF-κB phosphorylations in clasmatodendrosis (autophagic astroglial death) within the hippocampus of chronic epilepsy rats

Ji-Eun Kim,[1] Duk-Shin Lee,[1] Tae-Hyun Kim,[1] Hana Park,[1] Tae-Cheon Kang[1,*]

[1]Department of Anatomy and Neurobiology, Institute of Epilepsy Research, College of Medicine, Hallym University, Chuncheon 24252, Republic of Korea

* Correspondence to: T. -C. Kang, Department of Anatomy and Neurobiology, College of Medicine, Hallym University, Chuncheon, Kangwon-Do 24252, Republic of Korea; Tel: +82-33-248-2524; Fax: +82-33-248-2525; E-mail: tckang@hallym.ac.kr

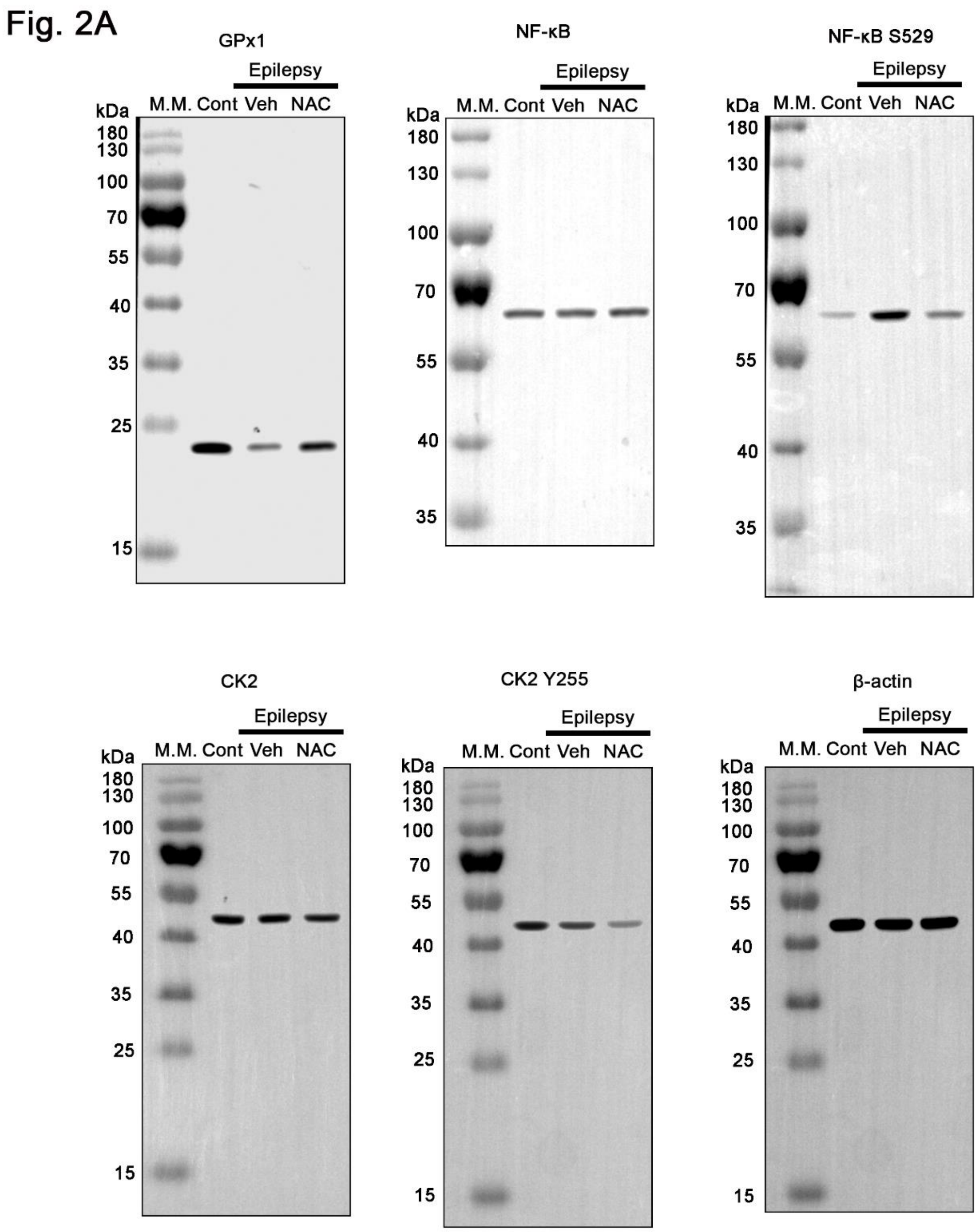

Supplementary Figure S1. Full-length gel images of Western blots in Figures 2A.

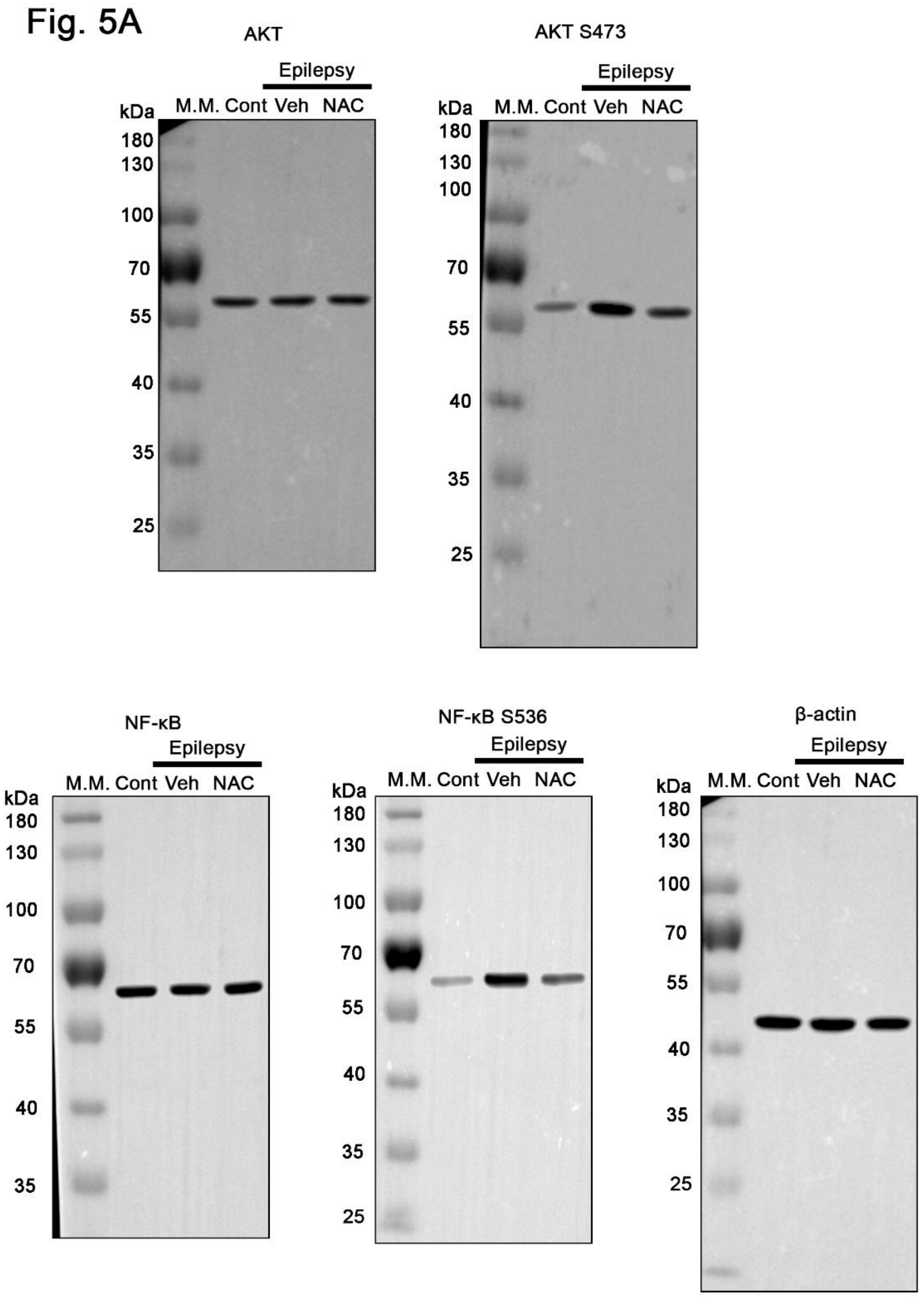

Supplementary Figure S2. Full-length gel images of Western blots in Figures 5A.

Fig. 8A

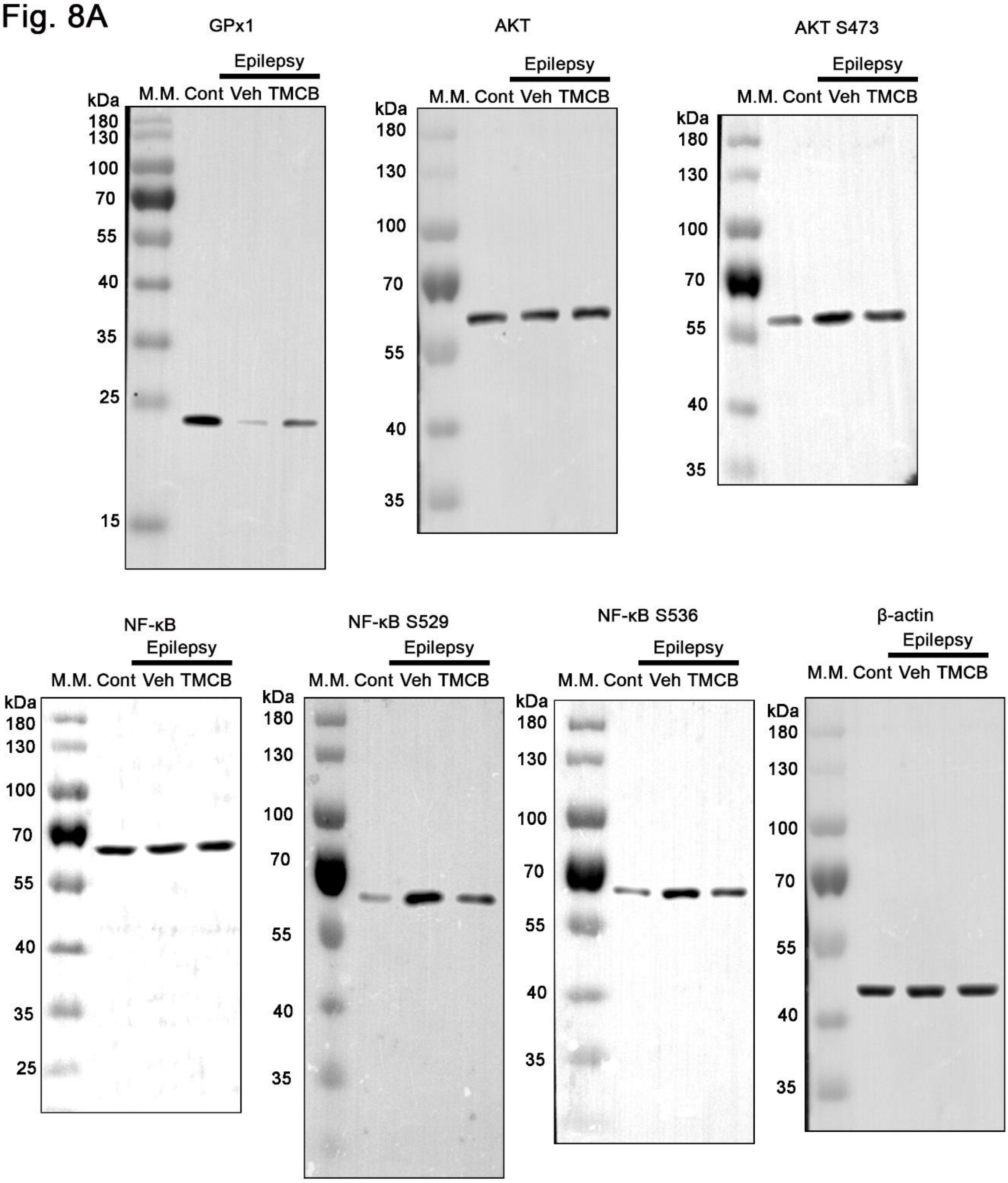

Supplementary Figure S3. Full-length gel images of Western blots in Figures 8A.

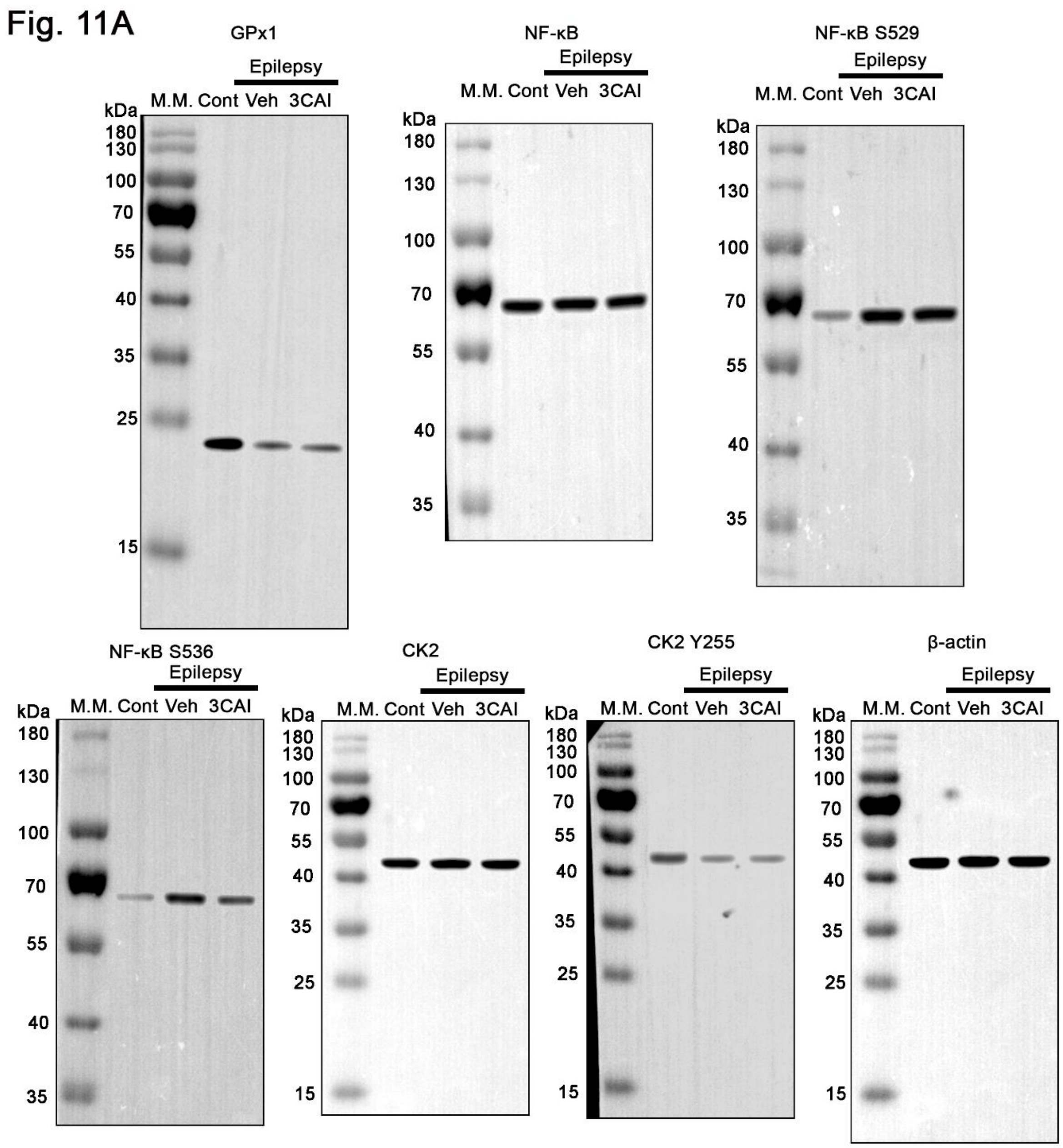

Supplementary Figure S4. Full-length gel images of Western blots in Figures 11A.
